# Nucleolar Division in the Promastigote Stage of* Leishmania major* Parasite: A Nop56 Point of View

**DOI:** 10.1155/2018/1641839

**Published:** 2018-10-10

**Authors:** Tomás Nepomuceno-Mejía, Luis Enrique Florencio-Martínez, Santiago Martínez-Calvillo

**Affiliations:** Unidad de Biomedicina, Facultad de Estudios Superiores Iztacala, Universidad Nacional Autónoma de México. Av. de los Barrios 1, Col. Los Reyes Iztacala, Tlalnepantla, Estado de México, CP 54090, Mexico

## Abstract

Nucleogenesis is the cellular event responsible for the formation of the new nucleoli at the end of mitosis. This process depends on the synthesis and processing of ribosomal RNA (rRNA) and, in some eukaryotes, the transfer of nucleolar material contained in prenucleolar bodies (PNBs) to active transcription sites. The lack of a comprehensive description of the nucleolus throughout the cell cycle of the human pathogen* Leishmania major* prompted us to analyze the distribution of nucleolar protein 56 (Nop56) during interphase and mitosis in the promastigote stage of the parasite. By* in silico* analysis we show that the orthologue of Nop56 in* L. major* (LmNop56) contains the three characteristic Nop56 domains and that its predicted three-dimensional structure is also conserved. Fluorescence microscopy observations indicate that the nucleolar localization of LmNop56 is similar, but not identical, to that of the nucleolar protein Elp3b. Notably, unlike other nucleolar proteins, LmNop56 remains associated with the nucleolus in nonproliferative cells. Moreover, epifluorescent images indicate the preservation of the nucleolar structure throughout the closed nuclear division. Experiments performed with the related parasite* Trypanosoma brucei* show that nucleolar division is carried out by an analogous mechanism.

## 1. Introduction

The cell nucleus contains a collection of nonmembrane-bound nuclear bodies (NBs) that participate in the regulation of essential functions, such as gene expression [1, 2]. The nucleolus is the most conspicuous NB that is present throughout the Eukarya domain [3, 4]. The fundamental role of the nucleolus is to coordinate ribosome biogenesis, an intricate multistep process that includes the transcription of ribosomal cistrons (rDNA) by RNA polymerase (RNA Pol) I and accessory factors, cleavage and chemical modification of precursor ribosomal RNA (rRNA), and assembly of mature rRNA species 18S, 5.8S, and 25/28S with numerous proteins and the 5S rRNA, product of RNA Pol III activity [5, 6].

The nucleolus is a dynamic organelle that is disassembled and assembled in organisms undergoing an open mitosis, such as human cells [7, 8]. The nucleolar cycle begins during the early stages of nuclear division, when several key nucleolar proteins involved in rDNA transcription and rRNA processing are negatively modulated by specific phosphorylation carried out by the cyclin B-dependent kinase 1 pathway [9–11]. Consequently, the rRNA synthesis is shut down and the nucleolar structure disappears. While proteins that participate in rDNA transcription remain attached to nucleolar organizer regions (NORs), rRNA processing proteins and small nucleolar RNAs (snoRNAs) as well as preserved pre-rRNAs localize to the cytoplasm and progressively accumulate along the entire periphery of condensed chromosomes, forming part of the perichromosomal compartment (PC) [12–15]. During chromosomal segregation, the components of PC migrate together with sister chromatids toward the poles of the mitotic spindle and remain associated with them until PC fragmentation. After that, the nucleolar material accumulates in intermediate nuclear structures called prenucleolar bodies (PNBs), before being released into transcriptionally active NORs, which are chromosomal loci where the synthesis and processing of rRNA have been reactivated. Restoration of ribosome biogenesis, close to the end of mitosis, triggers the nucleolar reassembly, a cellular process termed nucleogenesis [7, 8, 13, 16–24]. In* Saccharomyces cerevisiae*, an organism with closed mitosis, the nucleolus is located adjacent to the nuclear envelope, opposite to the spindle pole body. Unlike higher eukaryotes, the nucleolus persists during mitosis, and the duplicated nucleolus splits in two in early telophase, adopting symmetrical positions in mother and daughter nuclei [25, 26]. Given that the nucleolus is preserved, PNBs may not be formed during yeast mitosis, but to the best of our knowledge, this issue has not been addressed. However, as the nucleolus in this organism disassembles and reassembles during meiosis [26, 27], it is possible that PNBs might be involved in nucleolar assembly in this particular process.

Among the large number of components that are part of the nucleolar proteome, the nucleolar protein 56 (Nop56) is an essential factor highly conserved from Archaea to human that is actively involved in the biogenesis of the ribosomal subunits. It is one of the core elements of box C/D small nucleolar ribonucleoprotein particles (snoRNPs), which direct 2′-O- ribose methylation of specific residues in pre-rRNA [28–30] and are also involved in the endonucleolytic cleavages of the 35S rRNA primary transcript [31, 32]. In addition to Nop56, C/D snoRNPs contain a C/D snoRNA (like U3 or U14) and three other core proteins: fibrillarin, Nop58, and Snu13 [33].

In contrast to yeast and higher eukaryotes, little is known about structure and biogenesis of the nucleolus in the early-branched protozoan parasite* Leishmania*, the etiological agent of leishmaniasis, a significant public health problem in tropical and subtropical areas of the world.* Leishmania* is a member of the Trypanosomatidae family, which includes the pathogen parasites* Trypanosoma brucei* and* Trypanosoma cruzi*.* Leishmania* develops within phagolysosomes of infected macrophages as amastigotes and in the gut of the sandfly vector as extracellular promastigotes. The* L. major* genome possesses only ~12 copies of the rDNA unit per haploid genome, located on chromosome 27 as head-to-tail tandem arrays [34]. Synthesis and processing of rRNA are necessary steps for nucleolar building around the rDNA repeats grouped in transcriptionally active NORs. An ultrastructural analysis performed in* L. major* promastigotes showed that this parasite has a central, single, and spherical electro-dense nucleolus that, apparently, does not contain a fibrillar center [35].

Since Nop56 is an appropriate protein to investigate the process of nucleolar division, in this study we identified and analyzed the cellular location of the Nop56 orthologue in* L. major* (LmNop56). Bioinformatics analyses revealed that LmNop56 contains the three structural and evolutionary conserved domains and that its predicted three-dimensional structure is remarkably similar to that of the* S. cerevisiae* orthologue. By indirect immunofluorescence we showed that, in contrast to other nucleolar proteins, LmNop56 remains located in the nucleolus in aged cells. Moreover, our data showed that during interphase and closed mitosis LmNop56 persists and, seemingly, remains associated with the nucleolus. Interestingly, similar observations were obtained in procyclic* T. brucei* parasites.

## 2. Material and Methods

### 2.1. *In Silico* Analysis

Nop56 amino acid sequences of trypanosomatids, yeast, and human were obtained from TriTrypDB (http://tritrypdb.org/tritrypdb/) (release 36),* S. cerevisiae* genome (https://www.yeastgenome.org), and UniProtKB (https://www.uniprot.org), respectively. Multiple sequences alignments were performed with the Clustal Ω program (http://www.ebi.ac.uk/Tools/msa/clustalo/) and identical residues were colored manually. LmNop56 secondary structure determination was done using UCSF Chimera package (https://www.cgl.ucsf.edu/chimera/) and PSIPRED Protein Sequence Analysis Workbench (http://bioinf.cs.ucl.ac.uk/psipred/). Conserved domains were identified by Pfam (http://pfam.xfam.org), SMART (http://smart.embl-heidelberg.de), Prosite (http://prosite.expasy.org), and InterPro (https://www.ebi.ac.uk/interpro/) web pages. Three-dimensional homology models were obtained with SWISS-MODEL (https://swissmodel.expasy.org) and UCFS Chimera program [36] using the structure of* S. cerevisiae* (SWISS-MODEL Template Library ID: 5wyj.3.A) as a model.

### 2.2. Parasites

*L. major* promastigotes, strain MHOM/IL/81/Friedlin (LSB-132.1), were grown in BM medium (1× M199 medium pH 7.2 containing 10% heat-inactivated fetal bovine serum, 0.25× brain heart infusion, 40 mM HEPES, 0.01 mg/mL hemin, 0.0002% biotin, 100 IU/mL penicillin, 100 g/mL streptomycin, and 1× L-glutamine) at 28°C and harvested in the mid logarithmic (Log) or stationary (Sta) phases, four or seven days after initial inoculation. The* L. major* cell line that expresses a PTP-tagged version of the nucleolar protein Elp3b [37, 38] was maintained in BM medium with 50 *μ*g/mL G418. Procyclic parasites of the* T. brucei* strain 29-13 were cultured in SDM-79 medium supplemented with 10% fetal bovine serum at 28°C and harvested in the mid logarithmic phase. Epimastigotes of* T. cruzi* CL Brener strain were grown in LIT medium, as described elsewhere [39].

### 2.3. Western Blot Analysis

Trypanosomatid total protein extracts were solubilized in 5× Laemmli's buffer, fractionated by 10% SDS-PAGE and blotted onto a PVDF matrix. Western blot was performed using a polyclonal anti-LmNop56 mice serum [37] diluted 1:1000 in 2% nonfat dry milk prepared in phosphate buffered saline (PBS) containing 0.05% Tween-20. Antibody-antigen complexes were revealed by chemiluminescence, utilizing horseradish peroxidase-labeled goat anti-mouse IgG (BioLegend) and Immobilon™ Western kit (MILLIPORE). An *α*/*β*-tubulin polyclonal antibody (Cell Signaling Technology) was used as loading control.

### 2.4. Immunofluorescence Microscopy

Parasites were collected, rinsed twice with PBS, and attached onto poly-L-lysine-coated glass slides for 20 min at room temperature. Then, cells were fixed with 4% paraformaldehyde in PBS for 30 min at 4°C and permeabilized with 0.1% Triton x-100 in PBS for 10 min at room temperature. After several PBS washes, the unspecific binding sites were blocked with 2% bovine serum albumin (BSA) in PBS during 60 min. Preimmune or anti-LmNop56 immune sera were diluted in 1% BSA in PBS and incubated with the samples for 2 hours. After washing, goat anti-mouse IgG (H+L) antibody conjugated with Alexa Fluor® 488 dye (Molecular probes) was used. DNA was counterstained with propidium iodide and parasites were mounted with Vectashield®. For confocal microscopy, individual optical sections were obtained using a Carl Zeiss LSM 5 Pascal confocal laser microscope. Confocal micrographs were analyzed and prepared for presentation using the ImageJ processing program (https://imagej.nih.gov/ij/). On the other hand, in epifluorescence microscopy analysis, preparations of mid logarithmic and stationary phase parasites were coverslipped with Vectashield® mounting medium plus 4′,6-diamidino-2-phenylindole (DAPI; Vector Laboratories Inc.) after antibodies interaction. Visualization of fluorescent signal was carried out in a Carl Zeiss Axio Vert.A1 epifluorescence microscope. For double labeling experiments, cells were incubated overnight with 1% BSA in PBS containing a mix of anti-LmNop56 mice immune serum with (1) anti-histone H4 (Abcam), (2) anti-Prot C (for Elp3b-PTP) (Delta Biolabs), or (3) *α*/*β*-tubulin (Cell Signaling Technology) rabbit antibodies. LmNop56 was revealed by goat anti-mouse IgG (H+L) antibody conjugated with Alexa Fluor® 568 dye. Histone H4, Protein C-tag, and *α*/*β*-tubulin were visualized by goat anti-rabbit IgG (H+L) antibody coupled with Alexa Fluor® 488 dye. These samples were covered with antifading-DAPI solution, as described above. Elp3b distribution was analyzed in an* L. major* cell line where Elp3b was labeled with a PTP tag [37], using the anti-Prot C antibody in combination with an anti-*β*-tubulin antibody (Thermo Fisher). The Elp3b recombinant protein was observed using a goat anti-rabbit IgG (H+L) cross-adsorbed secondary antibody conjugated with Alexa Fluor® 594 dye. *β*-tubulin was visualized with goat anti-mouse IgG (H+L) antibody conjugated with Alexa Fluor® 488 dye. Parasite preparations were coverslipped with Vectashield® mounting medium plus DAPI, as indicated above. Epifluorescence micrographs were analyzed and prepared for presentation using the ZEN 2012 software (Blue edition).

## 3. Results

### 3.1. LmNop56 Is an Evolutionarily Conserved Protein

**Figure 1:**
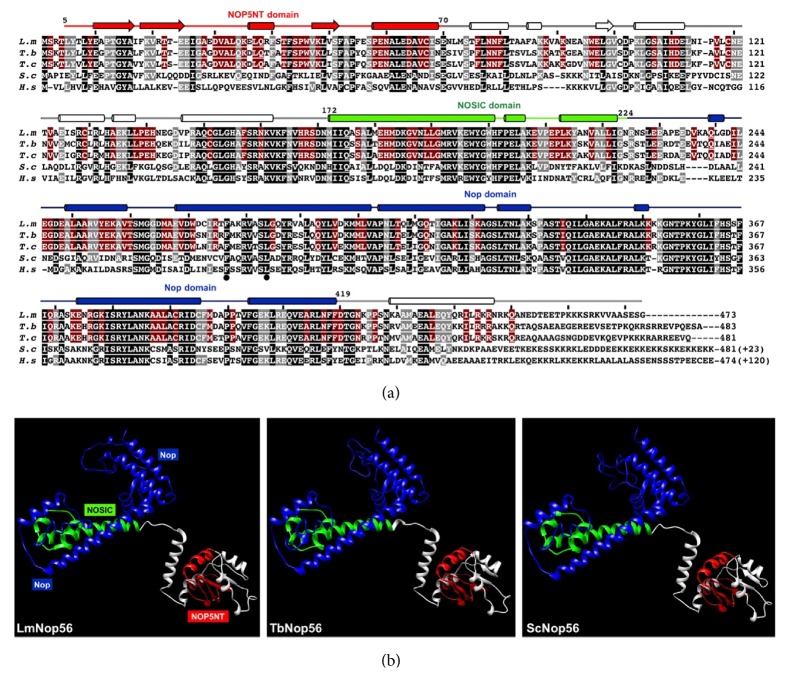
Sequence alignment and three-dimensional predicted structure of Nop56 orthologues. (a) Alignment of amino acid sequences of Nop56 from* L. major* (Lm; LmjF.10.0210),* T. brucei* (Tb; Tb927.8.3750)*, T. cruzi* (Tc; TCDM_07668),* S. cerevisiae* (Sc; YLR197W), and* H. sapiens* (Hs; O00567). Identical residues in all species are indicated by black shading, while conserved residues in four organisms are denoted by gray shading. Trypanosomatid-specific conserved residues are shaded in red. Predicted secondary structure elements in LmNop56 are displayed on top of the linear sequence. *β*-strands are symbolized by arrows and *α*-helices by cylinders. The NOP5NT, NOSIC, and Nop conserved domains are indicated in red, green, and blue, respectively. (b) The predicted three-dimensional modeling of Nop56 from* L. major* (residues 8 to 421),* T. brucei* (8 to 421), and* S. cerevisiae* (8 to 417) was obtained with the UCSF Chimera software using the SWISS-MODEL Template Library ID: 5wyj.3.A as a prototype. The three highly conserved domains are colored as indicated in panel (a).

In* L. major*, Nop56 is a 473 amino acid protein with a predicted molecular mass of 52.7 kDa, encoded by a single copy gene (ID: LmjF.10.0210) found on chromosome 10. Sequence analysis revealed that, like Archaea and eukaryotic orthologues, LmNop56 contains the three highly conserved domains termed NOP5NT (residues 5-70), NOSIC (residues 172-224), and Nop (residues 225-419) (Figure 1(a)). In other organisms, these domains are essential for the appropriate assembly and function of the box C/D snoRNPs. Multiple sequence alignments indicated that LmNop56 is ~80% identical to the* T. brucei* and* T. cruzi* orthologues, and 46 and 48% identical to Nop56 from human and yeast, respectively. The highest degree of primary structure conservation of Nop56 occurs within the Nop motif (Figure 1(a)). Although the sequence of the NOP5NT domain is the least conserved, it is predicted to fold into three *β*-sheets that are highly conserved across evolution (Figure 1(a), data not shown). The rest of LmNop56 mainly folds into *α*-helices dispersed throughout the protein. The three-dimensional structure for Nop56 from* S. cerevisiae* was recently obtained by cryo-electron microscopy, as part of the modeling of the entire 90S small subunit preribosome [40]. Homology modeling revealed that the hypothetical three-dimensional structure of LmNop56 (residues 8 to 421) is extensively similar to the reported yeast model, showing discrete N-terminal (NOP5NT) and C-terminal (Nop) domains (Figure 1(b)). The predicted structure for Nop56 from* T. brucei* is almost identical to the one obtained for LmNop56 (Figure 1(b)). Thus, the* in silico* analysis demonstrated that LmNop56 contains all the sequence and structural features that are present in Nop56 orthologues in other organisms.

### 3.2. LmNop56 Is a Nucleolar Component

**Figure 2:**
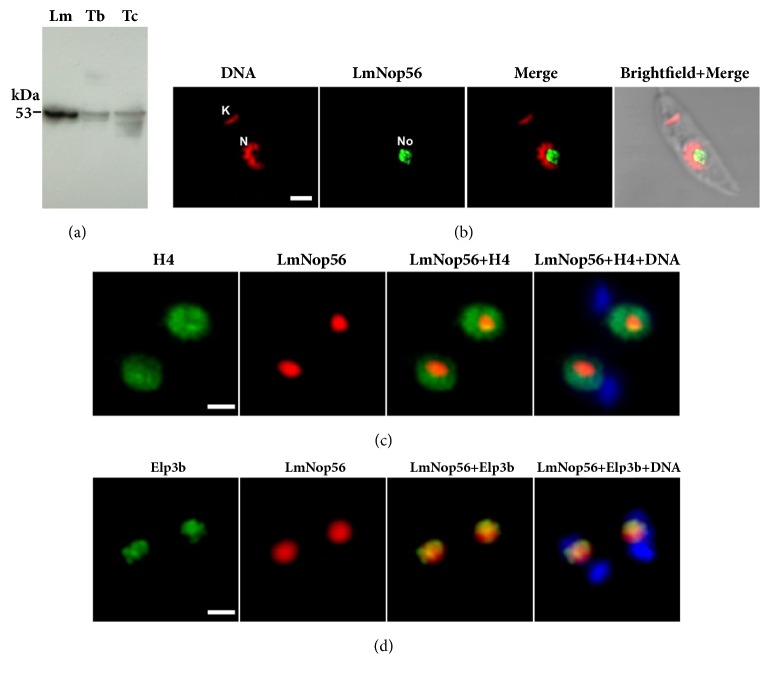
Nop56 expression in trypanosomatids and subcellular localization in* L. major*. (a) Western blot analysis of protein extracts from* L. major* promastigotes (Lm),* T. brucei* procyclic forms (Tb), and* T. cruzi* epimastigotes (Tc) was performed using an LmNop56 polyclonal antibody. (b) Indirect immunofluorescence experiment conducted with the same anti-LmNop56 serum and an anti-mouse IgG antibody conjugated with Alexa Fluor® 488 dye. Nuclei (N) and kinetoplast (K) in* L. major* promastigotes were counterstained with propidium iodide. Nucleolar (No) localization of LmNop56 was analyzed in single optical sections obtained by confocal microscopy. (c) Double indirect immunofluorescence assay carried out with antibodies raised against histone H4 and LmNop56. Histone H4 was revealed with anti-rabbit IgG coupled with Alexa Fluor® 488 (green), and LmNop56 with anti-mouse IgG conjugated with Alexa Fluor® 568 (red). (d) Double indirect immunofluorescence experiment performed with transgenic promastigotes expressing Elp3b-PTP. The antibodies employed were anti-LmNop56 and anti-Prot C. LmNop56 (red) was detected as indicated in panel (c), whereas Elp3b was revealed with anti-rabbit IgG coupled with Alexa Fluor® 488 (green). Images shown in panels (c) and (d) were obtained with an epifluorescence microscope; in these same panels, DNA was stained with DAPI (blue). Size bars represent 2 *μ*m.

To determine the expression of LmNop56 in* L. major* promastigotes, Western blot analysis was performed with a mouse polyclonal immune serum raised against the recombinant version of this protein [37]. A band of ~53 kDa was observed, which corresponds to the predicted size of LmNop56 (52.7 kDa) (Figure 2(a)). Notably, the polyclonal serum also recognized Nop56 in procyclic forms of* T. brucei* (54.3 kDa) and epimastigotes of* T. cruzi* (53.6 kDa) (Figure 2(a)). In order to determine the subcellular distribution of LmNop56, indirect immunofluorescence experiments were performed on fixed and permeabilized promastigotes using the anti-LmNop56 mice serum. Stained parasites were examined by confocal (Figure 2(b)) or wide-field optical epifluorescent microscopy (Figures 2(c) and 2(d)). This analysis clearly revealed a green fluorescent nuclear body located within a nucleoplasm region weakly stained with nucleic acid dye propidium iodide in actively replicating parasites, which might correspond to nucleolus of* L. major* (Figure 2(b)). A similar localization was observed by simultaneous labeling of Nop56 and histone H4 proteins, where the fluorescent red signal of LmNop56 is present within a specific region of the nucleoplasm (shown in green; Figure 2(c)). Nuclear and kinetoplast DNA are shown in blue. The nucleolar position of LmNop56 was confirmed by colocalization assays with the nucleolar protein Elp3b (Figure 2(d)). Elp3b is involved in Pol I transcription of rDNA in* T. brucei* [41] and colocalizes with 18S rRNA genes and with 5S rRNA in* L. major* [37]. While the majority of the signal overlaps (yellow spots in Figure 2(d)), some differences were observed in the nucleolar distribution of Nop56 and Elp3b. Therefore, these results demonstrate that LmNop56 is a nucleolar protein that partially colocalizes with Elp3b.

### 3.3. LmNop56 Is Concentrated in the Nucleolus and Additional Nuclear Regions in Nonproliferative Parasites

**Figure 3:**
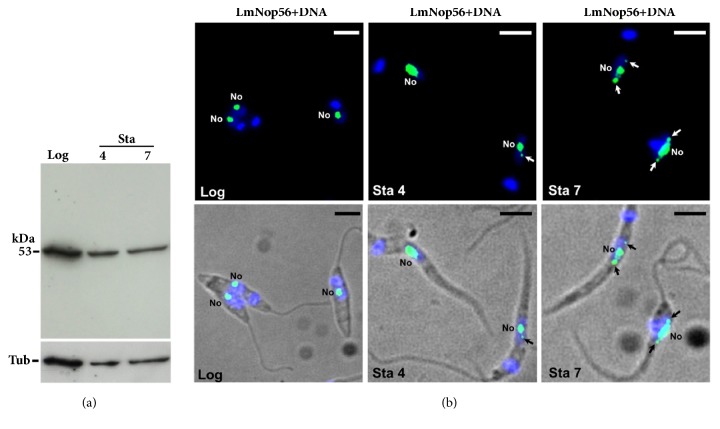
Expression and subcellular distribution of LmNop56 in* L. major* promastigotes in stationary growth phase. (a) Western blot analysis of total protein extracts from promastigotes harvested in the mid logarithmic phase (Log), and early (Sta 4) and late (Sta 7) stationary phases. The blots were probed with polyclonal LmNop56 immune serum and with *α*/*β*-tubulin antibody (loading control). (b) Indirect immunofluorescence assays performed with cells in Log, Sta 4, and Sta 7 phases using the anti-LmNop56 serum, and an anti-mouse IgG antibody conjugated with Alexa Fluor® 488 dye (green). Nuclear and kinetoplast DNA were counterstained with DAPI (blue). Nucleolar (No) and extra-nucleolar (arrows) green fluorescent signals were visualized with a conventional epifluorescence microscope. Size bars represent 5 *μ*m.

To investigate the presence of LmNop56 in quiescent promastigotes, we carry out a Western blot experiment with protein extracts obtained from parasites harvested in early (4 days, Sta 4) and late (7 days, Sta 7) stationary phases. As above, a single band with a molecular mass of around 53 kDa was observed in replicative parasites (Log; Figure 3(a)) and in nonproliferative cells (Sta 4 and 7; Figure 3(a)). The expression of LmNop56 was similar in growing and stationary phase cells, as indicated by the loading control with *α*/*β*-tubulin (Figure 3(a)). In order to determine the subcellular localization of LmNop56 in stationary growth phase promastigotes, indirect immunofluorescence assays were carried out. Quiescent organisms were visualized as thin and extended cells that possess an elongated nucleus and a long flagellum (Sta 4 and Sta 7 in Figure 3(b)). LmNop56 green signal was mainly located in the interior of the nucleolus (Figure 3(b)). However, spherical fluorescent foci located at the periphery of the nucleus were perceived in both early and late stationary growth phase promastigotes (Figure 3(b); white and black arrows). Hence, a portion of the LmNop56 protein delocalizes from the nucleolus to the nucleoplasm in nonproliferative parasites.

### 3.4. Nucleolar Distribution of LmNop56 during Mitosis

**Figure 4:**
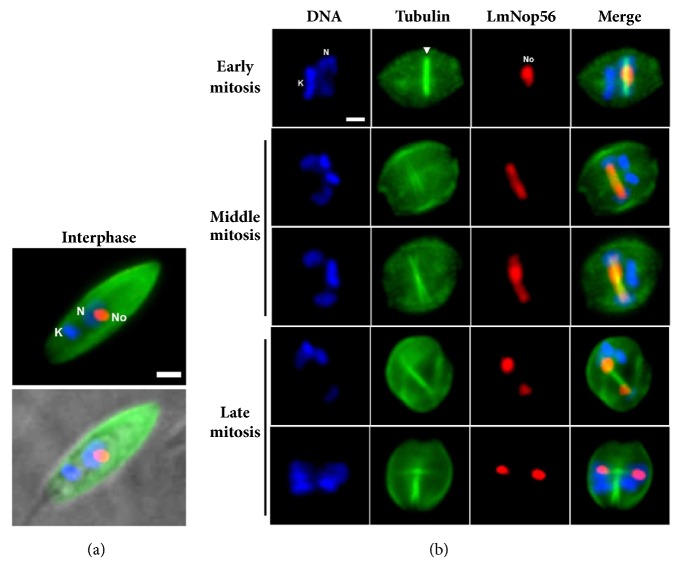
Intranuclear distribution of LmNop56 during cell division of* L. major*. Parasites fixed with paraformaldehyde were stained for double indirect immunofluorescence analysis using a mixture of primary antibodies against LmNop56 and *α*/*β*-tubulin (to label subpellicular microtubules and mitotic spindle) followed by anti-mouse IgG conjugated with Alexa Fluor® 568 (red) and anti-rabbit IgG coupled with Alexa Fluor® 488 (green) secondary antibodies. DNA was stained with DAPI (blue). (a) Interphase promastigote with (bottom image) and without (top image) brightfield. (b)* L. major* promastigotes at early, middle, and late stages of closed mitosis were analyzed. DNA, LmNop56, subpellicular microtubules, and mitotic spindle (indicated with a white arrowhead) are visualized. Colocalization of DNA and proteins is displayed in the Merge column. All images were obtained with a conventional epifluorescence microscope. K: kinetoplast; N: nucleus; No: nucleolus. Size bar denotes 2 *μ*m.

**Figure 5:**
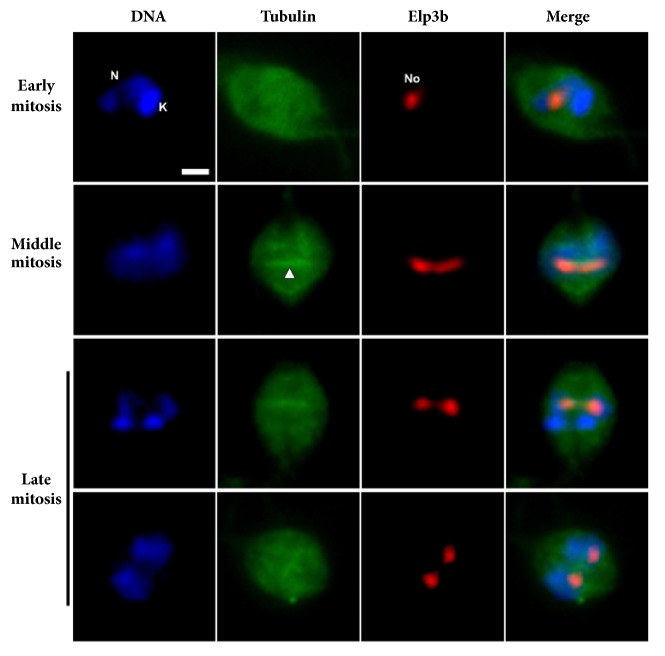
Nuclear distribution of Elp3b during the mitotic cell cycle of* L. major.* Promastigotes were fixed with paraformaldehyde and stained for double indirect immunofluorescence analysis using a mixture of primary antibodies against protein C (for recombinant Elp3b-PTP protein) and *β*-tubulin (to label subpellicular microtubules and mitotic spindle) followed by anti-rabbit IgG coupled with Alexa Fluor® 594 (red) and anti-mouse IgG conjugated with Alexa Fluor® 488 dye (green) secondary antibodies. DNA was stained with DAPI (blue).* L. major* promastigotes at early, middle, and late stages of closed mitosis were analyzed. DNA, Elp3b, subpellicular microtubules, and mitotic spindle (indicated with a white arrowhead) are visualized. Colocalization of DNA and proteins is displayed in the Merge column. All images were obtained with a conventional epifluorescence microscope. K: kinetoplast; N: nucleus; No: nucleolus. Size bar denotes 2 *μ*m.

To analyze the fate of LmNop56 during closed mitosis in* L. major* promastigotes, we performed double immunolabeling of LmNop56 and *α*/*β*-tubulin using a mixture of polyclonal antibodies raised against these proteins in fixed parasites. While the entire* L. major* body was illuminated by the green fluorescence of the subpellicular microtubules array, in interphase cells the red signal of LmNop56 is accumulated exclusively in the nucleolus (Figure 4(a)). In contrast to the nucleolar disassembly observed in other organisms at the beginning of open mitosis [8, 14], our micrographs suggest that in* L. major* the structure of the nucleolus is preserved throughout the nuclear division (Figure 4(b)). At the onset of closed mitosis, the nucleolar material (here represented by LmNop56) spreads in the central space of the elongated nucleus and interacts with the microtubules of the intranuclear mitotic spindle (Figure 4(b); early mitosis). As mitosis proceeds, LmNop56 progressively moves toward both ends of the nucleus, probably propelled by the driving forces of the spindle fibers and their associated motor proteins. This hypothesis is based on the marked colocalization found between LmNop56 and *α*/*β*-tubulin (Figure 4(b); middle mitosis). Finally, in late mitotic stages, the red fluorescent label of LmNop56 was localized only in a particular area of the nucleoplasm, which is poorly stained with DAPI (Figure 4(b); late mitosis). To further analyze the nucleolar division in* L. major*, we carried out double immunolabeling of Elp3b and *β*-tubulin. In this experiment we employed an* L. major* cell line where Elp3b was labeled with a PTP tag [37], using a mixture of antibodies that recognize the protein C epitope and *β*-tubulin. As shown in Figure 5, the distribution of the Elp3b signal is very similar to that observed with LmNop56, indicating that the nucleolus is preserved during the nuclear division. Thus, our results strongly suggest that during mitosis of* L. major* promastigotes the nucleolus persists and appears to separate out in a relatively intact form.

### 3.5. Fate of* T. brucei* Nop56 during Nuclear Division

**Figure 6:**
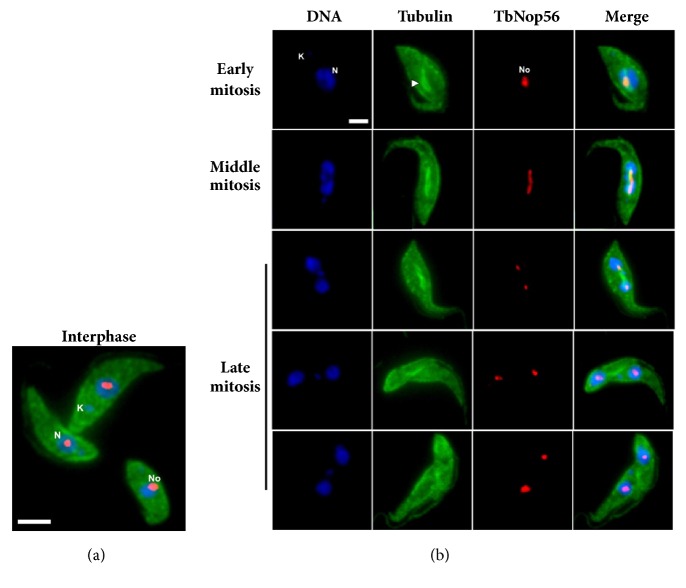
Subcellular localization of Nop56 during mitosis of procyclic* T. brucei* cells. Double Immunofluorescence assay was conducted in cells fixed with paraformaldehyde and then stained for Nop56 and *α*/*β*-tubulin (to detect subpellicular microtubules and mitotic spindle), followed by anti-rabbit IgG coupled with Alexa Fluor® 488 (green) and anti-mouse IgG conjugated with Alexa Fluor® 568 (red) secondary antibodies. Nuclear and kinetoplast DNA were counterstained with DAPI (Blue). (a) Procyclic parasites during interphase. Size bar indicates 5 *μ*m. (b) A set of representative micrographs of parasites in the different steps of closed mitosis is presented. Overlapping of DNA, tubulin, and TbNop56 is shown in the Merge column. Intranuclear mitotic spindle is indicated by a white arrowhead. Images were obtained with a conventional epifluorescence microscope. K: kinetoplast; N: nucleus; No: nucleolus. Size bar denotes 2 *μ*m.

To analyze the subcellular location of Nop56 in procyclic forms of* T. brucei*, indirect immunofluorescence experiments were performed using the antibody raised against LmNop56, which recognizes the* T. brucei* orthologue (Figure 2(a)). The cell bodies were stained by the green fluorescence of tubulin. Throughout the* T. brucei* cell cycle, Nop56 showed a subnuclear distribution pattern quite similar to that described in* L. major* (see Figures 2(b) and 4). During interphase, Nop56 (red) is located within a nuclear region weakly stained with DAPI (blue) that corresponds to the nucleolus (Figure 6(a)). In early stages of* T. brucei* mitosis, Nop56 was concentrated in a still spherical nucleolus (Figure 6(b); early mitosis). Gradually, the nucleus is extended, the mitotic spindle is assembled, and the nucleolar material (Nop56) is dispersed in the nucleoplasm, along the mitotic spindle (Figure 6(b); middle mitosis). At the end of mitosis, the fluorescent signal of Nop56 was located in the incipient nucleoli of both resultant cells (Figure 6(b); late mitosis).

## 4. Discussion

The nucleolus is a large membrane-less nuclear body where most steps of ribosome biogenesis take place. Two important events involved in maturation of pre-rRNA are nucleolytic cleavage of transcribed spacers and site-specific 2′-O-methylation of rRNA [5]. Both processes are directed by RNA-protein complexes formed by a box C/D snoRNA and four proteins known in human as 15.5K (Snu13p in yeast and L7Ae in Archaea), Nop56, Nop58, and the methyltransferase enzyme, fibrillarin (Nop1 in yeast). In Archaea, the Nop5 protein is a single homologue of eukaryotic Nop56 and Nop58 [28, 30, 31, 42]. Each box C/D snoRNP in eukaryotes contains a single snoRNA, two copies of 15.5K and fibrillarin, and one copy of Nop56 and Nop58 (which form a heterodimer). In Archaea, the Nop56/Nop58 heterodimer is replaced by a Nop5 homodimer.* In silico* analysis performed in the TriTrypDB database allowed us to identify the orthologue of Nop56 in* L. major*, which we characterized in this work. Trypanosomatids also contain orthologues of 15.5K and fibrillarin, but they do not seem to have a Nop58 orthologue. Consequently, similarly to Archaea, it would be expected that C/D snoRNPs in* L. major* and other trypanosomatids possess Nop56 homodimers instead of the typical Nop56/Nop58 heterodimers found in eukaryotes. Future studies will help to explore this hypothesis.

Nop56 is an evolutionarily conserved factor that orchestrates the correct assembly and functioning of snoRNPs, as it serves as a molecular bridge to bring together all the core components by means of its three well characterized modules. The assembly of box C/D snoRNPs has been extensively studied in Archaea, where they are known as box C/D sRNPs. The N-terminal motif, called NOP5NT, interacts with fibrillarin to form a catalytic heterodimer before joining, through the Nop domain, to L7Ae bound to guide sRNA [43, 44]. In parallel, two Nop5 proteins (each already attached to fibrillarin and L7Ae) homodimerize via the coiled-coil region of the NOSIC motif to complete the sRNP formation [43]. The NOP5NT, NOSIC, and Nop domains are cataloged as preserved domains in nucleolar proteins throughout evolution [45].

As shown by Western blot analysis, the molecular mass of Nop56 is conserved in trypanosomatids: ~52.7 kDa (473 aa) in* L. major*, ~54.3 kDa (483 aa) in* T. brucei*, and ~53.6 kDa (481 aa) in* T. cruzi* (Figure 2(a)). The orthologues in yeast (56.8 kDa, 504 aa) and human (66 kDa, 594 aa) are larger due to an extension in the C-terminal region (Figure 1(a)). Nevertheless, our results show that the overall sequence of Nop56 from* L. major* and other trypanosomatids is conserved and it contains the three characteristic Nop56 domains (NOP5NT, NOSIC, and Nop) (Figure 1(a)). Moreover, homology modeling revealed that the hypothetical three-dimensional structure of LmNop56 is very similar to the model of Nop56 reported for yeast (Figure 1(b)). Thus, the conservation of sequence and structure of LmNop56 strongly suggest that, similarly to other organisms, it may be involved in the assembly and function of box C/D snRNPs that participate in methylation [43, 44] and cleavage of the rRNA primary transcript [28–32].

Indirect immunofluorescence assays indicated that LmNop56 is a nucleolar protein, as the fluorescence signal was detected at the nuclear region less stained with propidium iodide (Figure 2(b)), histone H4 (Figure 2(c)), and DAPI (Figure 4(a)). Colocalization analysis with the nucleolar protein Elp3b proved that LmNop56 is located in the nucleolus (Figure 2(d)). The main areas of overlapping probably correspond to the fibrillar component of the nucleolus, since Elp3b regulates transcription of rDNA in* T. brucei* [41] and colocalizes with 18S rRNA genes in* L. major* [37]. The exclusive location of LmNop56 in the nucleolus is different from what has been reported in other organisms for several nucleolar proteins, including fibrillarin, that also localize to Cajal bodies [46]. Although Cajal bodies have not been reported in* Leishmania*, electronic microscopy data showed that the* T. cruzi* nucleus contains at least one Cajal body [47, 48].

Even though a small fraction of LmNop56 was observed outside the nucleolus in stationary phase promastigotes, most fluorescent signal was detected within a discrete nucleolus (Figure 3(b)). Thus, this data indicates that the nucleolus is preserved in nonproliferative* L. major* cells. This is different from* T. cruzi*, where the nucleolus is broken and disassembled in stationary phase epimastigotes; consequently, in aged* T. cruzi* epimastigotes, nucleolar proteins (such as Met-III and RPA31) are dispersed throughout the nucleoplasm [49, 50] or delocalized to the cytoplasm (fibrillarin) [51]. Changes in nucleolar structure and scattering of nucleolar proteins have also been observed in other organisms under nutrient starvation and inhibition of rDNA transcription, conditions that are present in stationary phase cultures. For instance, nitrogen deprivation in* S. cerevisiae* causes a reduction of nucleolar size accompanied by the nucleolar delocalization of RNA Pol I subunits A43 and A190, which became distributed throughout the nucleoplasm [52]. Similar results were obtained by rapamycin, an inhibitor of protein kinase TOR (target of rapamycin) involved in the regulation of rDNA transcription [52]. Also, repression of RNA Pol I transcription in HeLa cells produces segregation of the nucleoli and redistribution of nucleolar proteins B23 and nucleolin to the cytoplasm and the nucleoplasm, respectively [53]. The presence of LmNop56 in the nucleolus of quiescent cells is intriguing, considering that nonproliferative trypanosomatid cells show a reduced level of rDNA transcription [54, 55]. It is possible that LmNop56 remains associated with complete or partial snRNPs that would be ready to function when favorable growth conditions are reestablished or after differentiation to infective metacyclic promastigotes. Alternatively, in aged parasites LmNop56 might be involved in additional functions related to cell survival or stage transition.

Little attention has been paid to the division of the nucleolus at the end of mitosis in unicellular organisms. Based on the fact that Nop56 plays significant roles as a transacting element in ribosome biogenesis, we chose this protein as a target to analyze the nucleolar division in* L. major* and* T. brucei*, which undergo closed mitosis. To simplify the analysis, we divided the mitotic process into early, middle, and late mitosis, based on the distribution of nuclear and mitochondrial DNA, the mitotic spindle, and LmNop56. According to previous reports [56], early mitosis might correspond to prophase, middle mitosis to metaphase and anaphase, and late mitosis to telophase. When mitosis begins, Nop56 starts spreading in the middle part of the nucleus (Figures 4 and 6). As the cellular division advances, Nop56 is relocated to both ends of the elongated nucleus by interacting with the mitotic spindle, as suggested by their colocalization (Figures 4 and 6). In the end of mitosis, two new nucleoli are clearly observed in the still attached daughter cells. Notably, our data indicate that the nucleolus is preserved throughout the mitotic cell division of* L. major* promastigotes (Figures 4 and 5). Moreover, they support previous results that indicated the conservation of the nucleolus during mitosis in the insect stage of* T. brucei* (Figure 6) [57]. While early studies suggested that the nucleolus disappears when* T. cruzi* epimastigotes enter mitosis [58], a recent report showed that the nucleolus does not dissociate in the course of the cell division of this parasite [48]. Thus, nucleolar conservation during the mitotic cycle seems to be a distinctive feature in trypanosomatids. It would be important to determine whether transcription of rRNA genes remains active throughout mitosis in this group of organisms. Since our data strongly suggest that the nucleolus persists during the mitotic cycle of* L. major* promastigotes, the presence of PNBs would not be expected. The absence of PNBs during mitosis has been previously reported in* T. cruzi* epimastigotes [48] and* Giardia lamblia* trophozoites [59].

The nucleolus is a dynamic NB whose main function is the biosynthesis of ribosomes. However, this organelle appears to be involved in other transcendental cellular processes, including cell cycle progression and proliferation, apoptosis, senescence, telomerase activity, and the biogenesis of several ribonucleoprotein complexes [22, 60]. The plurifunctional nucleolus hypothesis [60] was reinforced by data of proteomic analysis that indicate that only ~30% of the nucleolar protein repertoire has a role in ribosomal biogenesis [22]. As in other eukaryotes, in* L. major* the most evident nucleolar activity is the synthesis of small and large ribosomal subunits. It remains to be determined if the nucleolus is involved in other relevant functions in this early-diverged eukaryote.

## 5. Conclusions

Our results showed that Nop56 is a structurally conserved protein found in the nucleolus throughout the cell cycle of* L. major* promastigotes and procyclic* T. brucei* cells. Contrary to what happens to nucleolar proteins from other eukaryotes, we found that LmNop56 remains mainly associated with the nucleolus in nonproliferative* L. major* parasites. We also observed that during closed mitosis the nucleolar structure, illuminated by Nop56 and Elp3b fluorescence, is preserved and inherited to daughter nuclei as a preassembled organelle pulled by the spindle fibers. Hence, we can speculate that during closed mitosis the rRNA processing factors (as LmNop56) are intimately linked to the nucleolus, probably in the form of RNP particles. Together, the findings reported in this manuscript significantly advance our understanding of the basic biology of the nucleolus in trypanosomatids, a group of early-branched eukaryotes.

## Data Availability

Images from replica experiments are available from the corresponding authors upon request.
